# Operative Versorgung von Fersenbeinfrakturen: Operationstechnik, Tipps und Tricks

**DOI:** 10.1007/s00113-026-01700-3

**Published:** 2026-03-18

**Authors:** Thomas Mendel, Christian Fischer, Philipp Kobbe, Sebastian Schilde

**Affiliations:** 1 Klinik für Unfall- und Wiederherstellungschirurgie, BG Klinikum Bergmannstrost Halle gGmbH, Merseburger Str. 165, 06112 Halle, Deutschland; 2 Department für Orthopädie, Unfall- und Wiederherstellungschirurgie (DOUW), Abteilung für Unfallchirurgie, Universitätsmedizin Halle, Ernst-Grube-Str. 40, 06120 Halle, Deutschland

## Lagerung

Für die operative Versorgung von Fersenbeinfrakturen bietet sich grundsätzlich die Verwendung einer Oberschenkelblutsperre an, insofern keine patientenspezifischen Kontraindikationen bestehen. Die Standardlagerung erfolgt in kontralateraler Seitenlage. Diese Position ermöglicht die optimale Frakturexposition, Ergonomie und Bildgebung. Das untere Bein wird leicht flektiert, das obere auf einem Kissen stabilisiert. Druckgefährdete Regionen (z. B. Fibulaköpfchen, Trochanter, Knieinnenseite) müssen gepolstert werden. Der ipsilaterale Arm auf der Tischseite wird auf einer Armlagerung nach vorn abgelegt oder hängend positioniert. Die simultane Versorgung von Fersenbeinfrakturen in Rückenlage ist aus Sicht der Autoren nicht geeignet, da die Nachteile einer erschwerten Zugänglichkeit und Bildwandlerdarstellung dem Vorteil des vermeintlich geringeren Aufwandes und des Zeitgewinns weit überwiegen.

Für ein geplantes mediales oder bilaterales Vorgehen bietet sich eine flexible Rückenlagerung des Patienten an, die eine ausreichende Innen- bzw. Außenrotation des Fußes erlaubt.

### Tipps und Tricks.

- Kniebeugung reduziert den Achillessehnenzug und erleichtert die Reposition des Tuber-Fragments.
- Carbon-Tischplatten ermöglichen uneingeschränkte Fluoroskopie und 3D-Bildgebung.

## Zugangswahl

**Abb. 1:**
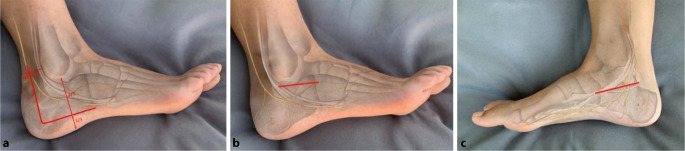
Schematische Abbildung der operativen Zugangswege (*rot*) zum Fersenbein mit Darstellung von knöchernen Strukturen, Sehnen und Nerven: **a** lateraler L‑förmiger Zugang, **b** Sinus tarsi-Zugang, **c** McReynolds-Zugang

Die Wahl des operativen Zugangs richtet sich nach Frakturtyp und Weichteilsituation. Drei etablierte Zugänge haben sich in der klinischen Praxis bewährt: der extensive laterale L‑förmige Zugang (ELA), der minimalinvasive Sinus-tarsi-Zugang und – zur Adressierung medial gelegener Frakturanteile – der mediale Zugang nach McReynolds (Abb. 1). Jeder dieser Zugänge bringt spezifische Vorteile, aber auch potenzielle Risiken mit sich (Tab. 1).

**Tab. 1:** Indikationsspektrum der 3 häufigsten Zugänge bei Fersenbeinfrakturen

Zugang	Typische Indikationen	Frakturklassifikation (z. B. nach Sanders)	Besonderheiten/Limitationen
Extensiver Lateraler L‑förmiger Zugang	Dislozierte intraartikuläre Frakturen mit komplexem Fragmentmuster – posterolaterale und zentrale Gelenkbeteiligung	Typen II–IV	Standardzugang bei komplexen Frakturen; hohes Risiko für Weichteilkomplikationen
Sinus-tarsi-Zugang	Gering dislozierte Gelenkfrakturen – extraartikuläre oder einfache intraartikuläre Frakturen	Typ II (Subtyp A/B), ausgewählte Typen III	Minimalinvasiv; sehr gute Sicht auf das Subtalargelenk von lateral, eingeschränkte Sicht auf mediale/anatomisch tiefe Gelenkflächen
Medialer Zugang (McReynolds-Zugang)	Beteiligung des Sustentaculum tali – medial dislozierte Frakturfragmente – Kombinationszugang bei Komplexfrakturen	Variabel; meist Ergänzung zu Typen III und IV	Zugang bei schwer erreichbaren medialen Fragmenten; selten isoliert verwendet

Sanders-Klassifikation: CT-basierte Einteilung der intraartikulären Calcaneus-Frakturen nach Anzahl und Lokalisation der Gelenkfragmente, Typen II–IV: zunehmende Komplexität mit multiplen Fragmenten und Beteiligung mehrerer Gelenkfacetten

### Extensiver lateraler L-förmiger Zugang

**Abb. 2:**
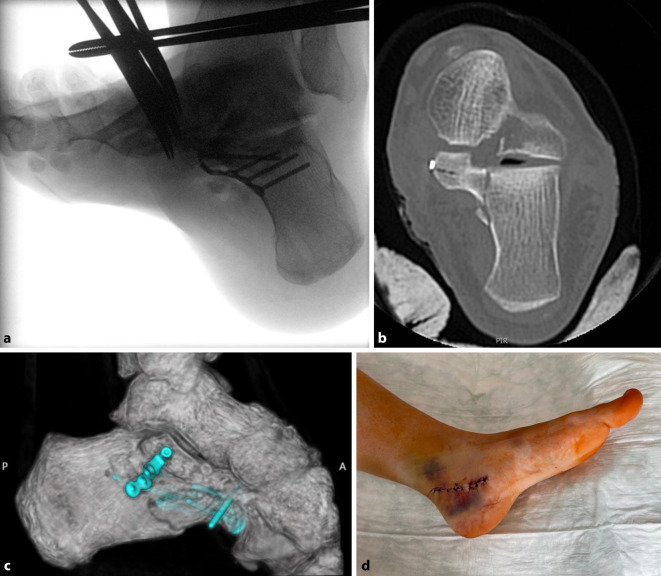
Schrauben- und Abstützplatten-Osteosynthese bei dislozierter Sustentaculum-Fraktur über einen McReynolds-Zugang. **a** Intraoperativer Broden-View, **b** paraaxialer CT-Schnitt auf Höhe des Sustentaculum tali, **c** 3D-CT-Rekonstruktion und **d** klinisches Bild des Zugangs nach Reposition und Osteosynthese

Der laterale L‑förmige Zugang stellt den Standardzugang zur operativen Versorgung komplexer, dislozierter, intraartikulärer Calcaneus-Frakturen dar. Die Hautinzision erfolgt in L‑Form (Abb. 2). Der senkrechte Schenkel verläuft auf Höhe der hinteren Drittelgrenze zwischen der Außenknöchelhinterkante und der Achillessehne. Der quere Schenkel verläuft auf der kaudalen Drittelgrenze entlang des lateralen Fersenrands am Übergang von der Leisten- zur Felderhaut nach anterior bis zur Basis des 5. Mittelfußknochens. Hierbei ist der M. abductor digiti minimi zu schonen. Der Weichteillappen wird unter Schonung des N. suralis, auf den im proximalen Wunddrittel zu achten ist, vollschichtig knochennah unter Durchtrennung des fibulokalkanearen Bandes präpariert. Der peronäale Sehnenkomplex wird am Trochlea peronealis abgelöst und im Lappen angehoben. Hierbei ist auf die Schonung des dorsal der Sehnen verlaufenden N. cutaneus dorsalis lateralis zu achten. Der Hautweichteillappen wird unter Eröffnung und Darstellung des Subtalargelenkes und des Sinus tarsi nach dorsal entwickelt und mit kräftigen K‑Drähten (z. B. 2,0 mm) im Talushals, im Processus anterior und posterior tali, schonend retiniert. Die laterale Kortikalis des Calcaneus, das Subtalargelenk, der Processus lateralis tali sowie das Tuber calcanei werden nun sichtbar. Mitunter werden die Präparation und Darstellung der Frakturzone erleichtert, wenn zuvor die Impaktion und Varusfehlstellung des Tuber-Fragmentes mit entsprechenden Repositionsmanövern korrigiert wird (siehe „Schritt 1: Reposition des Tuber-Fragmentes“).

### Sinus-tarsi-Zugang

Der minimalinvasive laterale Zugang wird vorwiegend bei weniger komplexen Frakturmustern eingesetzt, insbesondere bei Sanders-Typ II- und einigen Sanders-Typ-III-Frakturen, kann aber auch komplexere Frakturmuster adressieren. Er bietet eine hervorragende Sicht in das Subtalargelenk von lateral.

Die Hautinzision erfolgt über eine schräge Strecke von 3–4 cm zwischen dem lateralen Malleolus in Projektion auf die Basis des Os metatarsale 4. Nach Durchtrennen der Subkutis wird der N. cutaneus dorsalis lateralis identifiziert und geschont. Die Peronäalsehnen werden unter Eröffnung des distalen Retinaculums am Tuberculum peroneale ausgelöst und angeschlungen. Bei Bedarf wird die Sehne des M. extensor digitorum brevis mobilisiert. Anschließend wird das Lig. fibulocalcaneare durchtrennt und die Gelenkkapsel lateralseitig eröffnet. Die anterolaterale subtalare Gelenkfläche sowie die vordere Facette des Subtalargelenks werden dargestellt. Die laterale Wand des Calcaneus und der Gissane-Winkel sind in dieser Tiefe ebenfalls zugänglich. Vorbereitend für das Lager der Plattenosteosynthese erfolgt die knochennahe Präparation in Richtung Tuber calcanei. Je nach Frakturmuster kann der Zugang leicht nach dorsocranial oder distal erweitert werden.

### Medialer Zugang nach McReynolds

Der mediale Zugang nach McReynolds wird deutlich seltener verwendet, findet jedoch Anwendung bei Frakturen mit relevanter Beteiligung medialer Strukturen, insbesondere des Sustentaculum tali (Abb. 1).

Die Hautinzision verläuft bogenförmig entlang des medialen Fersenrands, beginnend knapp distal des Malleolus medialis über das Sustentaculum tali in Richtung mediale Plantarseite. Nach subkutaner Präparation werden N. tibialis posterior, die A. tibialis posterior und die V. saphena magna identifiziert und geschont. Die Sehnen des M. flexor hallucis longus und M. flexor digitorum longus werden nach posterior bzw. plantar retrahiert. So werden Sustentaculum tali und mediales Subtalargelenk direkt dargestellt. Die weitere knöcherne Präparation erfolgt entlang der medialen Calcaneus-Wand, ohne die neurovaskulären Strukturen zu kompromittieren.

## Reposition und temporäre Retention

Ziel ist die anatomische Rekonstruktion der subtalaren Gelenkfläche sowie der Form und Höhe des Fersenbeins unter Verwendung geeigneter osteosynthetischer Materialien.

### Schritt 1: Distraktion des Tuber-Fragmentes

**Abb. 3:**
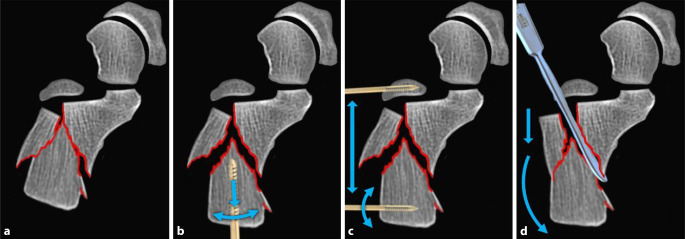
Techniken der Distraktion und Reposition des Tuber-Fragmentes mit jeweiliger Korrekturrichtung (*blaue Pfeile*): **a** impaktierter Ausgangssituation, **b** Westhues-Manöver mittels Schanz-Pin, **c** Distraktion über Distraktor und **d** Elevatorium

Im ersten Schritt erfolgt die Distraktion des Tuber-Fragmentes. Dieses große dorsale Hauptfragment befindet sich in der Regel in impaktierter und valgisierter Fehlstellung. Ziel dieser Maßnahme ist es, der Manipulation an den gelenkflächentragenden Fragmenten durch Aufhebung der Impaktion Freiraum zur ungehinderten Reposition zu schaffen. Mithilfe eines perkutan eingebrachten Steinmann-Pins mit T‑Handgriff gelingt eine kontrollierte Manipulation des Tuber-Fragmentes in Joystick-Technik (Westhues-Manöver, Abb. 3b; [2]). Die Korrektur des Böhler-Winkels kann dabei als intraoperative Referenzgröße genutzt werden. Das reponierte Tuber-Fragment wird temporär mit Kirschner-Drähten gegen den in der Regel stabilen medialen Sustentaculum-Block (Constant Fragment) fixiert.

Eine weitere Möglichkeit besteht in der Nutzung eines Elevatoriums, das entlang der typischerweise kraniolateral nach kaudomedial verlaufenden Frakturlinie eingebracht wird. Durch vorsichtiges Hebeln entlang dieser Spaltlinie kann das Tuber calcanei gezielt mobilisiert und gegen die mediale Wand reponiert werden (Abb. 3; [3]).

#### Tipps & Tricks.

Als Alternative zum Westhues-Manöver kann zur Reposition des Tuber-Fragmentes ein Distraktor verwendet werden. Lateral werden Kirschner-Drähte in den Processus lateralis tali und in das Tuber calcanei eingebracht. Die dreidimensional einstellbaren Achsen des Distraktors erlauben die kontrollierte Wiederherstellung der Fersenhöhe, die Korrektur der Rückfußdeformität sowie die Desimpaktierung der gelenkflächentragenden Fragmente. Der Distraktor ermöglicht eine stabile Fixation in korrigierter Stellung während der weiteren Rekonstruktion.

### Schritt 2: Rekonstruktion der subtalaren Gelenkfläche

Die Einsicht in das Subtalargelenk wird verbessert, indem das häufig lateral ausgeschlagene, dünnschalige Fragment („lateral bulge fragment“) türflügelartig aufgeklappt wird.

Die Reposition der Gelenkfläche orientiert sich am Constant Fragment. Dabei handelt es sich um das mediale, sustentakuläre Fragment, das aufgrund seiner ligamentären Verbindung zum Talus in der Regel in anatomischer Stellung verbleibt. Es dient als Referenz zur Rekonstruktion der posterioren Facette. Zeigt sich, dass dieses Fragment nicht formschlüssig zur talaren Gelenkfläche anliegt, sollte dieses zunächst reponiert und mit einem von plantar perkutan eingebrachten K‑Draht gegen den Talus fixiert werden.

Die gelenkflächentragenden Fragmente werden nun sukzessive „von innen nach außen“ an das Constant Fragment herangeführt und aufgebaut. Die korrekte Höhenlage, Gelenkflächenkongruenz und Fragmentrotation wird dabei mithilfe eines feinen Elevatoriums kontrolliert.

**Abb. 4:**
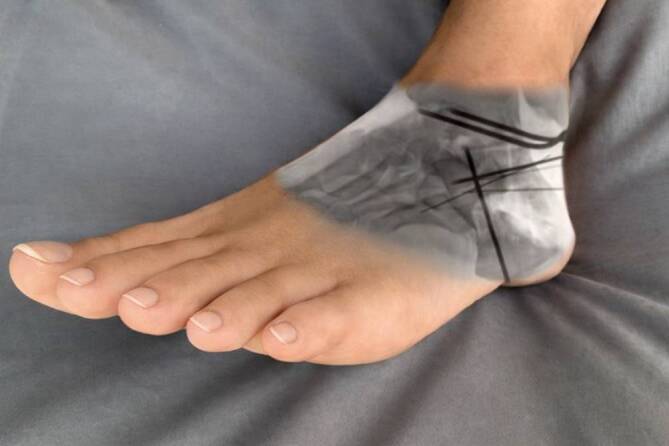
Fusionierte Bilddarstellung der Fußposition zur Darstellung der rekonstruierten posterioren Gelenkfacette im Broden-View

**Abb. 5:**
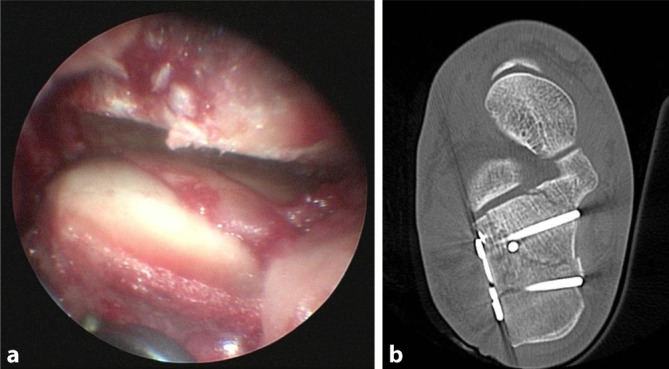
**a** Frakturoskopie, **b** postoperativer paraaxialer CT-Schnitt nach Versorgung einer Calcaneus-Fraktur Typ 2AC nach Sanders

Zur temporären Retention werden 1,4 oder 1,6 mm starke Kirschner-Drähte genutzt. Die Reposition kann über intraoperative Bildgebung in seitlicher Projektion sowie im Broden-View überprüft werden (Abb. 4). Optional kann auch eine Frakturoskopie zur direkten Kontrolle der Gelenkflächenkongruenz erfolgen (Abb. 5), wobei eine ausreichende Gelenkeinsicht unter Varusstress des Rückfußes gelingt.

#### Tipps & Tricks.

Bei komplexen, mehrfragmentären Frakturen (Typ III oder IV nach Sanders) mit einem oder mehreren zentralen Gelenkfragmenten kann zum schrittweisen Aufbau „von innen nach außen“ ein doppelt angespitzter K‑Draht von lateral kommend zunächst nach medial ausgebohrt werden, wobei die laterale Sitze auf Frakturniveau ohne Überstand zu liegen kommt. Nach Reposition des lateralen Fragmentes kann dieser Draht nun retrograd von medial nach lateral ausgebohrt werden und das laterale Fragment transfixieren.

**Abb. 6:**
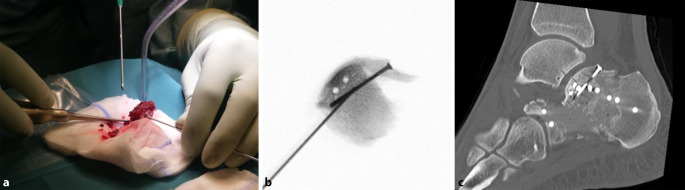
Ex-situ-Rekonstruktion der posterioren Gelenkfacette. **a** Intraoperative Situation, **b** situatives BV-Bild, **c** postoperatives CT

In Einzelfällen, z. B. bei irregulär verlaufenden intraartikulären Frakturlinien, kann eine Ex-situ-Rekonstruktion der dislozierten Fragmente erforderlich sein. Hier werden die zentralen Gelenkflächenfragmente sowie das laterale Gelenkfragment dem Situs entnommen, auf einem sterilen Tisch gesäubert und unter direkter Sicht anatomisch rekonstruiert (Abb. 6). Der rekonstruierte Gelenkblock wird anschließend wieder in den Situs eingebracht und exakt an das Constant Fragment reponiert. Diese Technik erlaubt auch bei schwer zugänglichen oder instabilen Fragmenten eine kontrollierte, anatomisch korrekte Wiederherstellung der Gelenkfläche.

**Abb. 7:**
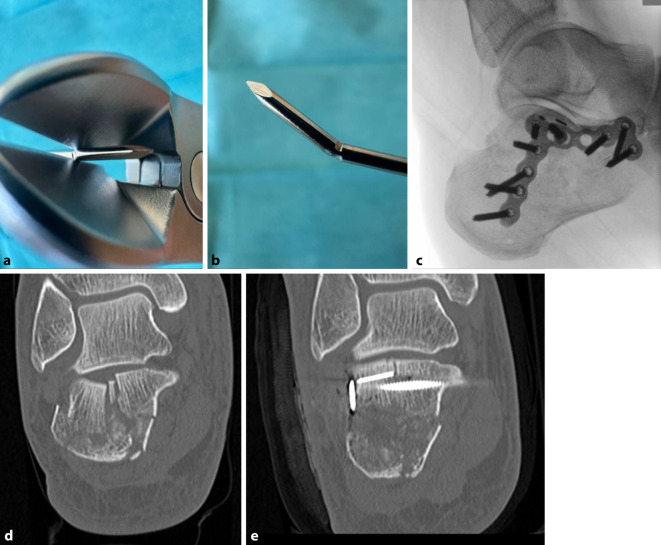
Beispielhafte Darstellung der „Lost-wire“-Anwendung mit **a** Präparation der Sollbruchstelle mittels Seitenschneider und **b** gezieltem Abbrechen der Drahtspitze nach Einbringung; **c** intraoperatives laterales BV-Bild mit „lost wires“ unterhalb der Gelenkfläche, **d** prä- und **e** postoperative koronare CT-Schnittbildebene

Auch „verlorene“ Implantate („Lost Wire“, „Lost Screw“) können beim schrittweisen Aufbau der posterioren Facette zur Stabilisierung gelenkflächentragender Zwischenfragmente Verwendung finden. Sie erlauben eine präzise Rekonstruktion, können bei etwaig notwendiger Materialentfernung jedoch nicht ohne größeren Flurschaden entfernt werden. Bei der Verwendung von K‑Drähten bieten sich die Durchmesser 1,4 und 1,6 mm an. Vor dem Einbringen des Drahtes kann mit dem Seitenschneider eine Sollbruchstelle erzeugt werden, sodass der bis dorthin eingebohrte Draht anschließend mit vorsichtigen Kippbewegungen ohne Überstand bündig zur Oberfläche abgebrochen werden kann (Abb. 7).

Bei stark dislozierten Frakturen vom Tongue type kann die anatomische Rekonstruktion der Gelenkfläche durch den Zug der Achillessehne erschwert sein [5]. In diesen Fällen kann das die Gelenkfacette und den Achillessehnenansatz tragende Tongue-type-Fragment mithilfe von 2 kräftigen K‑Drähten über das von Tornetta 1998 beschriebene „Essex-Lopresti-Manöver“ (ähnlich der Westhues-Technik) über eine nach distal rotierende Joystick-Bewegung direkt reponiert werden [6].

**Abb. 8:**
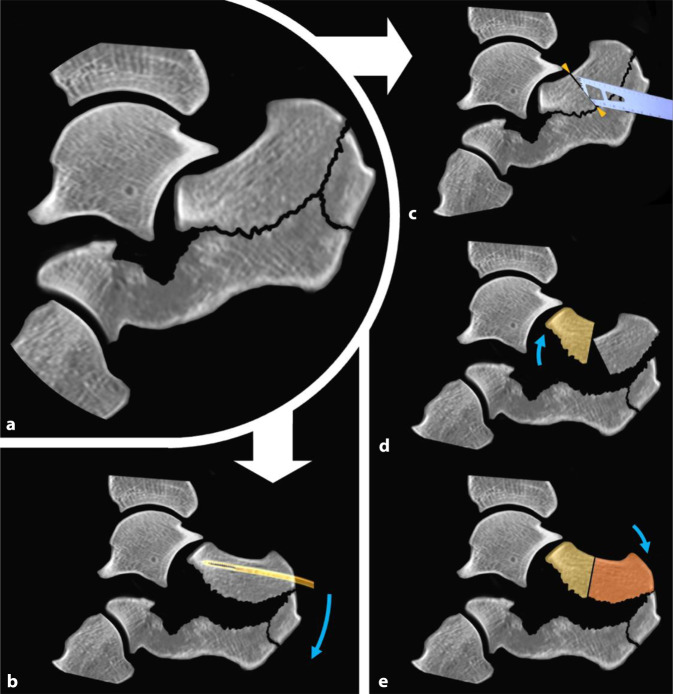
Repositionstechniken des Tongue-type-Fragmentes ausgehend von der **a** imprimierten Fraktursituation entweder als **b** Essex-Lopresti-Manöver mittels K‑Draht in Joystick-Technik oder mit **c** Osteotomie, gefolgt von der **d** Reposition des gelenkflächentragenden Fragmentes und dann **e** des den Ansatz der Achillessehne tragenden Fragmentes (*blaue Pfeile* – Repositionsrichtungen)

Gelingt dies nicht, bietet sich eine koronare Osteotomie des Tongue-type-Fragmentes dorsal der Gelenkfacette an. So kann zunächst die Gelenkfläche und anschließend das kraniale Tuber-Fragment reponiert werden (Abb. 8).

### Schritt 3: Rekonstruktion des Processus anterior calcanei

Dislozierte Frakturen des Processus anterior calcanei (v. a. bei Beteiligung des Kalkaneokuboidalgelenks) bedürfen ebenfalls einer Rekonstruktion. Für eine ausreichende Darstellbarkeit und Mobilität der Fragmente ist gelegentlich das Kerben des Ligamentum talocalcaneare interosseum erforderlich. Die typische kraniale Dislokation des Processus anterior sollte durch eine anatomische Rekonstruktion des Gissane-Winkels ausgeglichen werden. Hierdurch stellt sich eine etwaige Subluxation im Kalkaneokuboidalgelenk in der Regel indirekt spontan ein. Auch hier erfolgt abschließend die temporäre Fixation mit K‑Drähten.

Erst nach vollständiger Reposition aller relevanten Frakturkomponenten wird die definitive Fixation vorgenommen.

#### Tipps & Tricks.

Orientierende anatomische Landmarken für die Rekonstruktion der subtalaren Gelenkfläche:

- direkte Orientierung an der Gelenkfläche (nach Reposition oft nur noch erschwert einsehbar),
- die formschlüssige Reposition an die korrespondierende talare Gelenkfläche als „Template“,
- die passgenaue Position auf Höhe des Gissane-Winkels am Übergang zum Processus anterior calcanei.

### Schritt 4: osteosynthetische Stabilisierung

Intraartikuläre Fragmente werden je nach Größe mit interfragmentären Schrauben (2,0–3,5 mm) fixiert. Die definitive Stabilisierung erfolgt überwiegend durch laterale winkelstabile Plattenosteosynthesen. Bei minimalinvasivem Vorgehen werden zur Stabilisierung des Tuber-Fragmentes gegen den Gelenkblock bzw. den Processus anterior auch Großfragmentschrauben verwendet.

Alternativ kommen auch intramedulläre Nagelimplantate zur Anwendung.

Der Calcanail® (FH Ortho SAS, Heimsbrunn, Frankreich) wird über einen plantaren Zugang zum Tuber calcanei mit aufsteigender Trajektorie in Richtung der subtalaren Gelenkfläche eingeführt. Nach talokalkanearer Distraktion wird ein Arbeitskanal angelegt, der die indirekte Reposition der Gelenkfläche ermöglicht. Die anatomische Wiederherstellung von Rückfußhöhe, -länge und -achse erfolgt mittels Distraktor und indirekter Fragmenthebung über den Arbeitskanal (ggf. auch direkt über einen zusätzlichen Sinus-tarsi Zugang). Die Fixation der Fraktur wird durch den 2fach horizontal winkelstabil verriegelten Nagel erreicht. Über denselben Zugang kann zusätzlich eine primäre oder sekundäre subtalare Arthrodese mit einem längeren Nagelimplantat durchgeführt werden. Für den C-Nail (Medin, Nové Město na Moravě, Tschechien) erfolgt die direkte anatomische Rekonstruktion zunächst entsprechend den o.g. Schritten 1 bis 3 über einen Sinus-tarsi Zugang. Der Nagel wird anschließend unterhalb des Achillessehnenansatzes in Richtung der Mitte des calcaneocuboidalen Gelenkes implantiert und über einen Zielbügel mehrfach verriegelt.

**Abb. 9:**
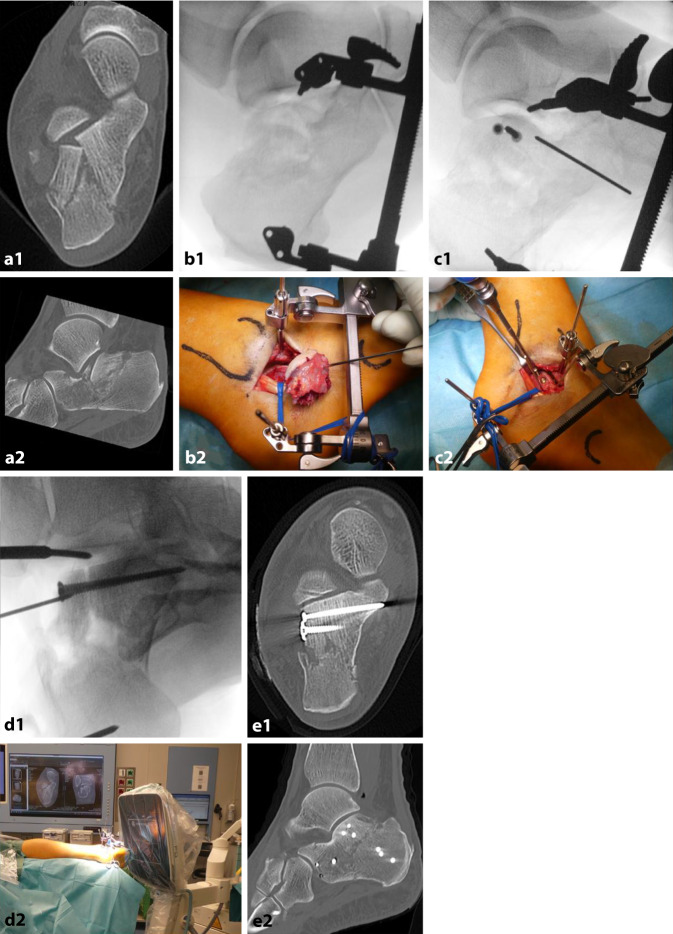
Klinischer Fall der Versorgung einer Calcaneus-Fraktur Typ IIA nach Sanders über einen Sinus-tarsi-Zugang mit primärer CT-Diagnostik in **a**_1_ parakoronarer und **a**_2_ sagittaler Rekonstruktion, Reposition des Tuber-Fragmentes mittels Distraktor im **b**_1_ seitlichen BV-Bild und **b**_2_ klinischem Situs mit Darstellung des lateralen Gelenkflächenfragmentes, Zugschraubenosteosynthese im **c**_1_ seitlichen BV-Bild bzw. im **d**_1_ Broden-View und jeweiliger klinischer Darstellung (**c**_2_ und **d**_2_) und postoperativem CT in **e**_1_ parakoronarer und **e**_2_ sagittaler Rekonstruktion

Abb. 9 dokumentiert das beschriebene Vorgehen an einem klinischen Fall.

## Wundverschluss

Im eigenen Vorgehen wird auf eine dezidierte elektrothermische Blutstillung wie auch auf die Einlage einer Drainage bewusst verzichtet. Die Blutsperre wird erst nach dem Wundverschluss und der Anlage eines Sterilverbandes mit elastischer Wickelung deaktiviert. Bei übungsstabiler Osteosynthese bedarf es postoperativ keiner zusätzlichen Ruhigstellung in Gips oder Cast.

## Nachbehandlung

In den ersten 3 Tagen ist eine konsequente Hochlagerung des Fußes erforderlich, um das Abschwellen der Weichteile zu unterstützen. Bei einer ausgeprägten postoperativen Schwellung kann Lymphdrainage angezeigt sein. Postoperativ erfolgt in den ersten 6 Wochen eine frühfunktionelle Beübung bei restriktiver Belastung von 0–20 kg. Um der Fibrosierungstendenz des Subtalargelenkes vorzubeugen, soll der Patient unter physiotherapeutischer Anleitung bereits ab dem ersten Tag zu regelmäßigen aktiven Kreiselbewegungen mit Extension und Flexion im oberen Sprunggelenk sowie Pro- und Supination im Subtalargelenk animiert werden, was zudem der Drainage der postoperativen Schwellung zuträglich ist [1]. Ist der Patient in der Lage, die Entlastung des Fußes im Sohlenkontaktlauf an Unterarmgehstützen sicher umzusetzen, sehen die Autoren keine Notwendigkeit einer zusätzlichen orthetischen protektiven Stabilisierung. Zudem sollte gezielt Augenmerk auf ein propriozeptives Training gelegt werden.

Ab der siebten Woche kann mit einer schrittweisen Belastungssteigerung von typischerweise 20 kg/Woche begonnen werden. Mit Erreichen der Vollbelastung liegt der Fokus auf der Gangschulung sowie auf Kraft- und Koordinationsübungen. Leichtere körperliche Belastungen sind in der Regel nach 3 bis 4 Monaten möglich, schwere körperliche bzw. sportliche Beanspruchung jedoch erst nach 6 bis 12 Monaten.

## Fazit für die Praxis

- Dislozierte intraartikuläre Frakturen mit einer Gelenkstufenbildung ≥ 2 mm sowie Deformation des Rückfußes stellen eine Indikation zu offener Rekonstruktion und Stabilisierung dar.
- Das Verständnis der Anatomie und Biomechanik des Rückfußes ist Voraussetzung für eine erfolgreiche Osteosynthese, weshalb diese Operationen erfahrenen Fußtraumatologen vorbehalten bleiben sollten.
- Die Frakturmorphologie und -komplexität bedingen die Zugangswahl: > 90 % lateral, einfache Typ-II(A/B)- und ausgewählte Typ-III-Frakturen über Sinus-tarsi-Zugang, komplexe Typ-III-/Typ-IV-Frakturen über erweiterten L‑förmigen Zugang, mediale Fragmente des Sustentaculum über McReynolds-Zugang.

## References

[1] Amlang MH, Rammelt S (2016) Calcaneal nail C‑nail. Unfallchirurg 119(3):239–244 26780911 10.1007/s00113-015-0138-0

[2] Westhues H (1935) Eine neue Behandlungsmethode der Calcaneusfrakturen. Zugleich ein Vorschlag zur Behandlung der Talusfrakturen. Zentralbl Chir 35:995–1002

[3] Eastwood DM, Langkamer VG, Atkins RM (1993) Intra-articular fractures of the calcaneum. Part II: Open reduction and internal fixation by the extended lateral transcalcaneal approach. J Bone Joint Surg Br 75(2):189–195 8444935 10.1302/0301-620X.75B2.8444935

[4] Rammelt S, Gavlik JM, Barthel S, Zwipp H (2002) The value of subtalar arthroscopy in the management of intra-articular calcaneus fractures. Foot Ankle Int 23(10):906–916 12398142 10.1177/107110070202301004

[5] Essex-Lopresti P (1952) The mechanism, reduction technique, and results in fractures of the os calcis. Br J Surg 39(157):395–419 14925322 10.1002/bjs.18003915704

[6] Tornetta P 3rd (1998) The Essex-Lopresti reduction for calcaneal fractures revisited. J Orthop Trauma 12(7):469–473 9781770 10.1097/00005131-199809000-00007

